# Upcycling Biodegradable PVA/Starch Film to a Bacterial Biopigment and Biopolymer

**DOI:** 10.3390/polym13213692

**Published:** 2021-10-27

**Authors:** Brana Pantelic, Marijana Ponjavic, Vukasin Jankovic, Ivana Aleksic, Sanja Stevanovic, James Murray, Margaret Brennan Fournet, Jasmina Nikodinovic-Runic

**Affiliations:** 1 Institute of Molecular Genetics and Genetic Engineering, University of Belgrade, Vojvode Stepe 444a, 11000 Belgrade, Serbia; branapantelic@imgge.bg.ac.rs (B.P.); vukasinjankovic@imgge.bg.ac.rs (V.J.); ivana_aleksic@imgge.bg.ac.rs (I.A.); 2 Institute of Chemistry, Technology and Metallurgy, University of Belgrade, Njegoseva 12, 11000 Belgrade, Serbia; mponjavic@ihtm.bg.ac.rs (M.P.); sanjas@ihtm.bg.ac.rs (S.S.); 3 Athlone Institute of Technology, Dublin Road, Athlone, N37HD68 Co. Westmeath, Ireland; jmurray@ait.ie (J.M.); mfournet@ait.ie (M.B.F.)

**Keywords:** biopolymers, thermoplastic starch, mechanical properties, PVA, biodegradation, upcycling, biopigments

## Abstract

Meeting the challenge of circularity for plastics requires amenability to repurposing post-use, as equivalent or upcycled products. In a compelling advancement, complete circularity for a biodegradable polyvinyl alcohol/thermoplastic starch (PVA/TPS) food packaging film was demonstrated by bioconversion to high-market-value biopigments and polyhydroxybutyrate (PHB) polyesters. The PVA/TPS film mechanical properties (tensile strength (*σ*_u_), 22.2 ± 4.3 MPa; strain at break (*ε*_u_), 325 ± 73%; and Young’s modulus (*E*), 53–250 MPa) compared closely with low-density polyethylene (LDPE) grades used for food packaging. Strong solubility of the PVA/TPS film in water was a pertinent feature, facilitating suitability as a carbon source for bioprocessing and microbial degradation. Biodegradability of the film with greater than 50% weight loss occurred within 30 days of incubation at 37 °C in a model compost. Up to 22% of the PVA/TPS film substrate conversion to biomass was achieved using three bacterial strains, *Ralstonia eutropha* H16 (*Cupriavidus necator* ATCC 17699), *Streptomyces* sp. JS520, and *Bacillus subtilis* ATCC6633. For the first time, production of the valuable biopigment (undecylprodigiosin) by *Streptomyces* sp. JS520 of 5.3 mg/mL and the production of PHB biopolymer at 7.8% of cell dry weight by *Ralstonia eutropha* H16 from this substrate were reported. This low-energy, low-carbon post-use PVA/TPS film upcycling model approach to plastic circularity demonstrates marked progress in the quest for sustainable and circular plastic solutions.

## 1. Introduction

Upcyclable products meet the conditions required for circularity by being indefinitely recyclable, without reduction in value or usability. The cyclical repurposing of plastic resources, as opposed to contributing to recalcitrant waste stockpiles, is essential to achieving a sustainable socio-economic ecosystem. Progressing plastics circularity requires minimizing polluting factors and resource loss while facilitating continuous material repurposing. Circularity and regenerative cycles are fundamental to the Earth’s natural ecosystems. In nature, examples of the biodegradation and bioregeneration of processes for natural polymers and end-of-life bio-based materials abound. In applications spanning from medical devices to food packaging, biodegradable blends and their composites are playing an increasing role in mitigating against negative environmental impacts [1]. Active developments are ongoing to overcome biodegradable plastics performance shortcomings such as brittleness, gas-barrier properties, and processability [1,2]. In addressing the considerable challenge of circularity, it is vital that post-use regeneration routes for these new plastics are identified to secure resource and value continuity throughout the repurposing/revalorization process [3,4].

Starch can be obtained from the waste streams and by-products of multiple renewable food sources, including corn and potatoes [5,6]. Thermoplastic starch (TPS) is a relatively low-cost material that lacks rigidity and mechanical strength [1,7]. Conversion of starch to a thermoplastic material can be achieved by adding small-molecule plasticizers such as glycerol, sorbitol, and urea [8], or by blending with other polymers such as polycaprolactone (PCL), polylactic acid (PLA), or polyvinyl alcohol (PVA and PVOH) [9,10]. A recent advancement in starch modification allowed its application as a water treatment agent [11].

PVA is a water-soluble synthetic polymer with good barrier properties afforded by its polar hydroxyl groups. PVA is highly processable, but brittle, and its biodegradation has been repeatedly demonstrated [12]. When compounded together, PVA and TPS have been shown to form blends with barrier and mechanical parameters suitable for food packaging applications, while also being biodegradable. Cost-competitive PVA/TPS blends have been demonstrated as alternatives to the widely used recalcitrant food packaging barrier layer plastic, ethylene vinyl alcohol (EVOH) [13,14]. The current cost of starch-based packaging films is not cost-competitive compared to petroleum-based plastics; however, starch blends significantly reduce the costs of bio-based biodegradables and, with the rising costs of petroleum, may be competitive in the near future [15]. Potential applications in food packaging and recent advances in the production of starch-based films are high and have been reviewed recently [16]. For comparison, the current cost of PVA/TPS pellets used in this work is between EUR 3/kg and 4/kg, while the cost of PHB is estimated to be between EUR 10/kg and 24/kg which is still multiple times more than petroleum-based plastics such as PET [17]. As the plastics market and value chain move toward increased sustainability and circularity, the cost of biopolymers, including PVA, TPS, and blends thereof, furcate to strongly decrease in cost as production levels greatly increase.

Investigation into the biochemical pathways of PVA degradation started as early as the 1970s. The proposed mechanism of degradation involves two enzymes, both isolated from *Pseudomonas* species. First, an oxidase (a secondary alcohol oxidase [18] or a specific PVA oxidase [19]) leads to the formation of *β*-hydroxyketone groups, which are later cleaved by the second enzyme, a *β*-diketone hydrolase [18]. A number of *Pseudomonas* strains have been identified as PVA degraders [20], along with *Bacillus* [21,22] and several fungal yeast strains [12].

TPS has been reported to be completely biodegradable under all environmental conditions [23]. It has been shown that starch films degrade completely in compost at 60 °C within 30–84 days [24,25]. More recently, the degradation of PVA/TPS blend films by two strains isolated from compost has been reported. Strains *Bacillus* sp. DG22 and *Paenibacillus* sp. DG14 were found to degrade the films to a high degree (45–75% depending on the type of blend used) [21]. The degradation of neat PVA films has been reported to be less than 20% [26] with the increased amenability degradation as part of a blend in keeping with literature reports.

Polyhydroxyalkanoates (PHA) are another type of biodegradable and sustainable polymer that possesses properties similar to petroleum-based polymers [27]. PHAs can be obtained by the fermentation of sustainable feedstocks such as food waste [28]. *Ralastonia eutropha* H16 (*Cupriavidus necator* H16) is a well-known producer of the PHA homopolymer polyhydroxybutyrate (PHB) and is able to convert a variety of substrates [29]. In the search for low-cost substrates, PHB-producing bacteria *Azotobacter chroococcum* and *Haloferax mediterranei* were able to produce PHB using starch as the sole carbon source [30]. *R. eutropha* has been shown to efficiently produce PHB from waste potato starch [31]. To the best of our knowledge, no reports on the production of PHB from PVA have been reported. While the upcycling of packaging materials to biodegradable plastics has been attempted previously, obtaining bacterial biopigments from packaging materials is a novel concept. Bacterial pigments have been attracting attention as natural colorants, which are a safer and more sustainable alternative to synthetic colorants with applications in food and pharma industries [32].

In this work, PVA/TPS films (in a 1:1 ratio), targeted for packaging applications, were produced by extrusion and characterized. The application of this film as a carbon substrate for the production of value-added microbial biopigments compounds such as undecylprodigiosin [33], and biopolymers such as polyhydroxyalkanoates (specifically PHB), was demonstrated. In addition, the biodegradation of this material was assessed in a compost model system at 37 °C under different conditions. In the quest for sustainable and circular plastic solutions, an array of new polymer blends and composites that encompass upcycling capacities are being developed.

## 2. Materials and Methods

### 2.1. Materials and Chemicals

Extruded films of 0.01–0.02 mm in thickness were made from ‘Biosol’ pellets (B000129EP) and were supplied by EcoBioCroatia Ltd. (Zagreb, Croatia). ‘Biosol’ pellets contain: 50–60% PVA, 30–40% TPS, 10–20% glycerol, and 1–5% coconut oil, according to the manufacturer’s specifications.

Inorganic salts (K_2_SO_4_, Na_2_HPO_4_ × 12H_2_O, KH_2_PO_4_, NH_4_Cl, MgSO_4_ × 7H_2_O, and CaCl_2_ × 2H_2_O), PVA (87–90% hydrolyzed, average molecular weight 30,000–70,000), and solvents were purchased from Sigma-Aldrich (Munich, Germany). Microbiological media and components such as yeast extract, tryptone, and casamino acids were sourced from Oxoid (Thermo Fisher Scientific, Cambridge, UK).

### 2.2. ATR-Infrared Spectroscopy (ATR-FTIR)

The Fourier transform infrared spectroscopy of PVA/starch films before and after degradation was recorded using an IR-Affinity spectrophotometer (Thermo Scientific, NICOLET iS10, Waltham, MA, USA). The spectra were collected in the range from 4000 to 400 cm^−1^ at room temperature with a scan step of 4 cm^−1^, and 32 scans were carried out in total.

### 2.3. Differential Scanning Calorimetry and Thermogravimetric Analysis (DSC/TG)

Coupled differential scanning calorimetry (DSC)/thermogravimetric (TG) analysis was performed on a TA Instruments SDT Q600 instrument (TA instruments Ireland, Dublin, Ireland). Analysis of the starting PVA/TPS film was measured over a temperature range from 25 °C to 900 °C, at a heating rate of 10 °C/min, in a nitrogen atmosphere. The weight of each sample was approximately 3 mg. 

### 2.4. Atomic Force Microscopy (AFM) Analysis

The surface morphology was investigated by atomic force microscopy (AFM) with a NanoScope 3D (Veeco, Plainview, NY, USA) microscope operated in contact mode under ambient conditions. Silicon nitride probes with a spring constant of 0.07–0.4 Nm^−1^ were used. Image analysis was carried out using Nanoscope image processing software. ‘Top-view’ images display the selected image from a top-down perspective, while height information is represented by the color at a given point. ‘Surface Plot’ images display the selected image with color-coded height information in a two-dimensional perspective.

The surface roughness value (RMS) was calculated as the root-mean-square average of height deviations taken from the mean data plane, as given by Equation (1):

(1) $$\sqrt{\frac{\sum Z_{i}^{2}}{n}}=R_{q}$$

where *Z_i_* is the maximum vertical distance between the highest and the lowest data points in the image.

### 2.5. Mechanical Properties of Films

The tensile mechanical properties of cast PVA/starch films were evaluated at ambient temperature using a Shimadzu Autograph AGS-X (Kyoto, Japan) servo-hydraulic universal test machine, equipped with a 1 kN load cell. A crosshead speed of 5 mm/min was used for testing and stress was applied until complete fracture of the samples occurred. Five samples in the form of filmstrips (75 mm × 15 mm × 0.01 mm) were used for testing. 

Tensile tests were also carried out on extruded PVA/TPS films (in a 1:1 ratio) using an Ametek TA-1 texture analyzer (Berwyn, IL, USA). The machine was equipped with a 20 N load cell and tests were run using a crosshead speed of 5 mm/min. Due to the anisotropic nature of the extruded film, testing was carried out on samples cut in both the extruded (longitudinal) and perpendicular (transverse) directions. The film, with a thickness of ~0.02 mm, was cut using a die with a width of 5 mm and total length of 100 mm, which resulted in a gauge length of 33 mm when placed between the grips. Soft rubber strips were placed between the sample and stainless-steel grips to reduce local stress concentrations in samples and prevent premature grip failure. 

For all tensile tests, the average values ± standard deviation of the tensile strength, strain-at-break, and Young’s modulus were reported. The Young’s modulus values were calculated in the 0–5% strain region, where elastic behavior occurred in all cases.

### 2.6. Light Fastness

Optical properties of the film samples before and after degradation under different composting conditions were determined by measuring percent transmittance using a UV-Vis spectrophotometer (UV-1800, Shimadzu, Japan). The film samples were cut into rectangular pieces and placed directly in the side of spectrophotometer magnetic cells using air as a reference with a wavelength range of 200–900 nm [34]. Transparency of the films was tested by measuring the percentage transmittance at 260 nm, and the average values of percent transmittance of three samples for each film was presented.

### 2.7. Study of Films Water-Contact Properties

#### 2.7.1. Swelling Index (*Q* %)

The swelling index, *Q*, was investigated in phosphate-buffered solution (PBS) over 60 min, at room temperature, according to the procedure adopted from Costa et al. [35]. PVA/TPS packaging samples, with dimensions of 50 mm × 15 mm × 0.01 mm, were dried in an oven at 105 °C until a constant mass was reached, after which the samples were placed in vials containing 50 mL of PBS solution. After 60 min, the films were collected, the excess PBS was removed using absorbent paper, and the films were weighed. The increase in mass was presented as swelling index (*Q* %) according to Equation (2):

(2) $$Q\;\%=\frac{m_{s}-m_{0}}{m_{0}}\times 100$$

where *m_s_* is the mass of the swollen film and *m*_0_ is the initial mass of the dry film. The experiment was performed in triplicate and presented as an average value ± standard deviation (SD).

#### 2.7.2. Solubility (*ML* %)

The solubility of PVA/TPS commercial material was determined by employing a procedure described by Flores et al. [36] with adaptation as follows: films (50 mm × 15 cm × 0.01 mm) were dried in an oven at 105 °C prior to immersion in 50.0 mL of PBS. The vials were kept with constant stirring at room temperature for 48 h. The solubility index indicated the mass lost (*ML* %) by each sample and was calculated according to Equation (3):

(3) $$ML\;\%=\frac{m_{1}-m_{0}}{m_{0}}\times 100$$

where *m*_1_ is the final mass of the dried sample after immersion in PBS and *m*_0_ is the starting mass of the sample. Each measurement was performed in triplicate and the results were expressed as the average value ± standard deviation (SD).

#### 2.7.3. Water Uptake (*WU* %)

Starch and PVA are highly hygroscopic materials. The moisture content of the PVA/TPS films was investigated by conditioning the samples at room temperature (20–25 °C) in desiccators at controlled humidity levels using saturated potassium sulfate solution (98% K_2_SO_4_ as a humectant). The experiment was performed in triplicate, and the water uptake was tracked over seven days. The water uptake, *WU %*, was calculated according to the following equation:

(4) $$WU\;\%=\frac{W_{t}-W_{i}}{W_{i}}$$

where *W_t_* represents the weight after immersion at a predetermined time *t* at a 98% relative humidity of the films, and *W_i_* refers to the initial weight of dry film [37].

### 2.8. Upcycling and Biodegradation Assessment

#### 2.8.1. PVA/TPS Film as Substrate for Bacterial Growth

Bacterial strains (*Bacillus subtilis* ATCC6633, *Streptomyces* sp. JS520 [38], and *Ralstonia eutropha* H16 (*Cupriavidus necator* ATCC 17699) [39]) were grown in tryptone soy broth overnight at 30 °C and 180 rpm. These starter cultures were centrifuged (10,000 rpm/20 min/4 °C), washed with 5 mL of PBS, and used for inoculation (10%, *v*/*v*).

The films were rinsed with ethanol and dissolved in hot (~90 °C) sterile H_2_O (40 g/L). This solution was used as a carbon source. To a Mineral Salt Medium (MSM; 9 g/L Na_2_HPO_4_ × 12H_2_O, 1.5 g/L KH_2_PO_4_, 1 g/L NH_4_Cl, 0.2 g/L MgSO_4_ × 7H_2_O, 0.2 g/L CaCl_2_ × 2H_2_O, 0.1% trace elements solution, and 0.025% N-Z amine) [40], PVA/TPS solution was added to final concentrations of 20 g/L and 10 g/L. In order to test the potential of these microorganisms to utilize PVA as a sole carbon source, MSM was supplemented with filter-sterilized PVA in 5 g/L and 10 g/L final concentrations.

Erlenmeyer flasks (100 mL volume) containing 20 mL of media were inoculated with 2 mL of washed-overnight cultures and further incubated for 14 days at 30 °C/180 rpm. At the end of incubation, cultures were centrifuged (5000 rpm/20 min/4 °C), the bacterial pellet was dried for 3 days at 65 °C, and the weight was determined.

From the *Streptomyces* sp. JS520 culture grown on PVA/TPS films, undecylprodigiosin was extracted and quantified spectrophotometrically as described previously [38]. A wavelength scan from 200 to 800 nm was taken (Spectrophotometer Ultrospec 3300pro, Amersham Biosciences, Amersham, UK) and the absorption maxima at 533 nm were used for calculation.

From the *R. eutropha* H16 culture grown on PVA/TPS films, PHB was quantified by gas chromatography as described previously from the dried biomass, where 8–10 mg of dried cells was methanolized and the PHB monomers were extracted in chloroform [41].

#### 2.8.2. Model-Compost Degradation

Biodegradation of the PVA/TPS films in model compost was carried out at a constant temperature of 37 °C for 30 days using the protocol described by Ponjavic et al. [42] (Figure S1). The model compost consisted of a commercial mixture of raw materials used for the cultivation of white button mushroom (80%, *w*/*w*) and commercial universal soil for garden and potting (20%, *w*/*w*), both from ACS Garden, Belgrade, Serbia. Pieces of film (3 × 5) cm^2^ (weighing ~38 mg) were placed into a Petri dish and buried in compost, and changes in film appearance were compared by taking photographs.

Three types of compost were used: nontreated compost, heat-pretreated (120 °C, 20 min), and bioaugmented compost. The bioaugmented compost was enriched with log_10_ 4 cells (*R. eutropha* H16, *Streptomyces* sp. JS520, and *B. subtilis* ATCC6633) per gram of compost and mixed thoroughly with a sterile spatula. A fresh aliquot of bacterial cultures was added after 2 weeks, and the appropriate amount of sterile water was added every 5 days to ensure constant humidity. Film samples were removed, washed with cold water, air-dried, and photographed on days 6, 10, 20, and 30. Samples were removed on days 10 and 30 for further analysis and weight measurements.

## 3. Results and Discussion

### 3.1. Thermal and Mechanical Properties of PVA/TPS Films

**Figure 1:**
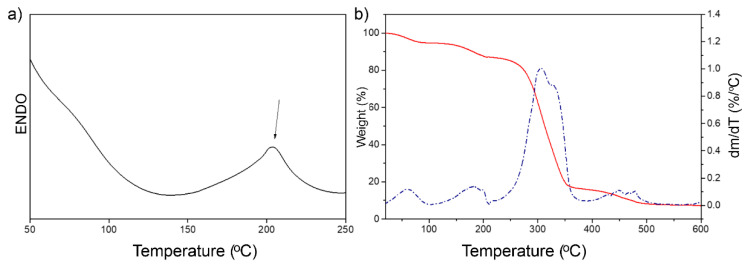
Representative (**a**) DSC thermogram and (**b**) TG/DTGA curves for tested PVA/TPS films.

Thermal properties of the PVA/TPS film material are presented in Figure 1, where a representative DSC thermogram and TGA curve are shown. DSC analysis indicated one endothermic, melting peak at 203.6 °C, coming from the PVA, which is in agreement with data from the literature for films prepared from PVA/TPS [43], while the heat melt, ∆*H*_m_, was 74.3 J/g. From the TGA/DTG plots, it was evident that the degradation of the film occurred in a few steps, as four characteristic peaks were detected. The first two degradation steps, where less than 20 weight % was lost, occurred between 20 and 180 °C, and represented the evaporation/dehydration, due to the PVA/TPS known high hydrophilicity and tendency to adsorb moisture from the environment.

The main degradation peak at the temperature range from 306 to 332 °C was associated with the degradation of PVA and TPS. The first stage of PVA degradation commenced at 306 °C, while the degradation of TPS commenced at 332 °C. The overlapping of the two main degradation temperatures is due to the compatibility between PVA and starch, where a more thermally stable cyclic hemiacetal in starch improves thermal stability within the PVA/TPS blends [44]. The DTG results indicated that the maximum degradation temperature was at 306 °C when the first degradation step of PVA occurred. This corresponds to the elimination of low-molar-mass carbon-based molecules that are products from the breakage of the polymer carbon-carbon backbone associated with the elimination of hydroxyl groups from dehydration reactions [45]. The second degradation step of PVA occurred at the higher temperature range from 450 to 477 °C and was attributed to the thermal decomposition of high-molar-mass polyenes that are derived during the first stage of degradation [46]. An amount of 90% of weight was lost at 460 °C, and the residue at 500 °C was 8.3 wt%. In the case of TPS, the thermal degradation peak due to decomposition appeared as a ‘shoulder’ of the PVA main degradation peak at 332 °C, when breaking the starch structure with CO and CO_2_ elimination, and the formation of carbonaceous residues occurred [47].

**Figure 2:**
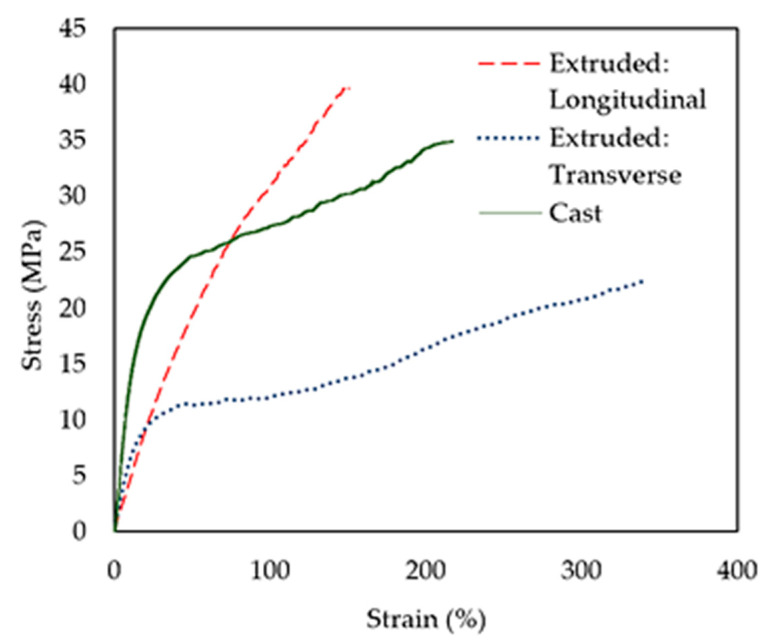
Mechanical stability of PVA/TPS films.

Mechanical properties of the cast PVA/TPS films used for the composting degradation experiments were determined in terms of tensile strength (*σ*_u_), strain at break (*ε*_u_), and Young’s modulus (*E*). Representative stress–strain curves are presented in Figure 2 and a summary of the results is given in Table 1.

Within blends, PVA facilitates good mechanical properties by interfacial adhesion between the PVA and different polymers used to prepare PVA blend films [48]. Blending PVA with TPS has the potential to decrease the mechanical properties, including tensile strength and strain at break. The investigated PVA/TPS cast films showed an average strain at break (*ε*_u_) value of 197% and tensile strength (*σ*_u_) of 37.2 MPa, which are higher compared to previously reported PVA/TPS blend films of the same composition (1:1) [49]. The even distribution of TPS strands is one of the most important factors that dictates the mechanical properties of PVA/TPS blend films. High plastic deformation and increased TPS content could promote agglomeration and void formation within the polymer matrix [48], impeding interfacial adhesion between the two components and subsequently decreasing the tensile strength [50]. The high mechanical performance of the tested PVA/TPS films can hence be attributed to the excellent distribution of the TPS polymer chains within the PVA polymer matrix, providing good interfacial compatibility, and the smooth surface of the films, as was observed by both AFM and SEM analysis (PVA/TPS control).

The longitudinal samples demonstrated almost purely elastic behavior with an average tensile strength *σ*_u_ of 38.6 MPa (Figure 2). The transverse samples demonstrated very different behavior with a limited elastic region up to around 6–10 MPa, followed by a large plastic area, and failure occurring at an average strength of 22.2 MPa. The elastic regions were similar at earlier stages of testing between the longitudinal (average E of 60.3 MPa) and transverse (average *E* of 52.9 MPa) cases. However, as elongation continued, the transverse samples deformed plastically, demonstrating high strains-to-break (average *ε*_u_ of 325%) compared to the longitudinal samples (average *ε*_u_ of 136%). The degree of anisotropy demonstrated from tensile testing is typical for extruded films, due to chain alignment in the extruded (longitudinal) direction during processing. 

All measured mechanical properties of the tested materials are similar to those reported in the literature for PVA/TPS films [49]. The properties compare well with polyethylene films, whose properties are shown for comparison in Table 2. The low modulus and high strength of the PVA/TPS material mean that it is extremely flexible while maintaining the load-bearing capacity required in demanding applications.

PVA/TPS films indicated a high swelling index, *Q*, after only 60 min of immersion in PBS due to the highly hydrophilic nature of both PVA and starch polymers, while the solubility index, *ML*, was calculated to be 7.5, which meant that after 48 h in PBS, 7.5% of polymer films was dissolved in the medium. 

**Figure 3:**
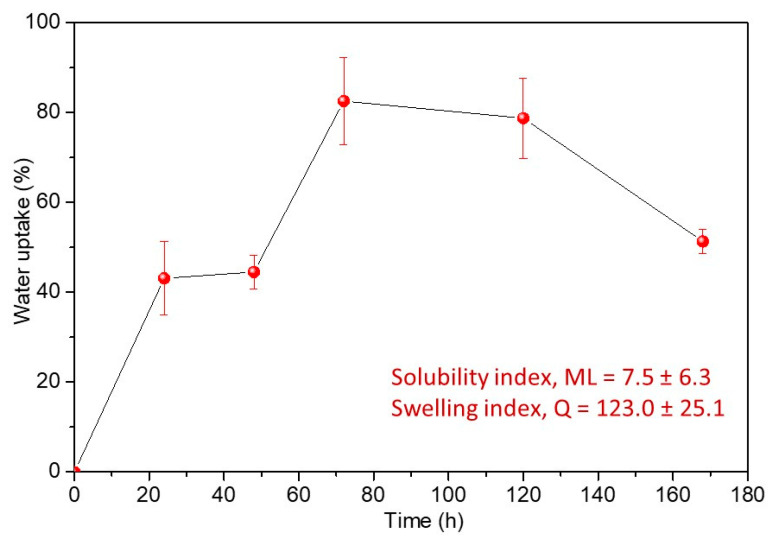
Water uptake of investigated PVA/TPS films over seven days.

Water uptake was followed by immersion in a saturated solution of potassium sulfate over seven days, and the results are presented in Figure 3. In the starting 48 h of immersion, approximately 40% of moisture was absorbed, while a significant increase in moisture uptake was detected after 72 h of exposure (80%). After 72 h, the water uptake started to decrease, probably due to the dissolution of highly hygroscopic PVA/TPS material. A water uptake of less than 60% was calculated after seven days of exposure.

The water absorption is a very important property of biopolymers as it directly affects their degradability, and the study of water uptake is essential especially in relation to food packaging applications. Most biodegradable polymers possess a high sensitivity to water absorption. Once the water is absorbed into the polymer, it makes this material suitable for microorganisms to grow, and the fungi and bacteria can utilize the polymer as an energy source [50,54]. Both PVA and starch are highly hydrophilic polymers due to the free –OH groups that form hydrogen bonds with water [8]. However, after the blending of PVA and starch, the decrease in water uptake was observed in comparison to neat PVA and starch as a consequence of hydrogen bonding between PVA and starch, which further affects the quantity of free –OH groups, causing the decrease in water sensitivity [8,55]. Finally, the investigated PVA/TPS films with a water uptake of more than 80% after 3 days of exposure to moisture appeared as a highly hydrophilic material that would be easily degraded by microorganisms and used as a bacteria energy source. 

### 3.2. Bacterial Upcycling and Biodegradation of PVA/TPS Material

The upcycling of a variety of carbon-rich waste streams including nonoxygenated PE and PP wax into PHA has been achieved [56,57]. Postconsumer PET has been successfully upcycled to PHA and a novel bio-based poly(amide urethane) (bio-PU) recently [58]. In this study, the upcycling of PVA/TPS material to bacterial biopigments and PHB was assessed. PVA/TPS film was cut into strips, comprehensively characterized by various techniques, and used for biotechnological upcycling and biodegradation assessment.

#### 3.2.1. PVA/TPS Films and PVA as Carbon Source for Bacterial Growth

**Figure 4:**
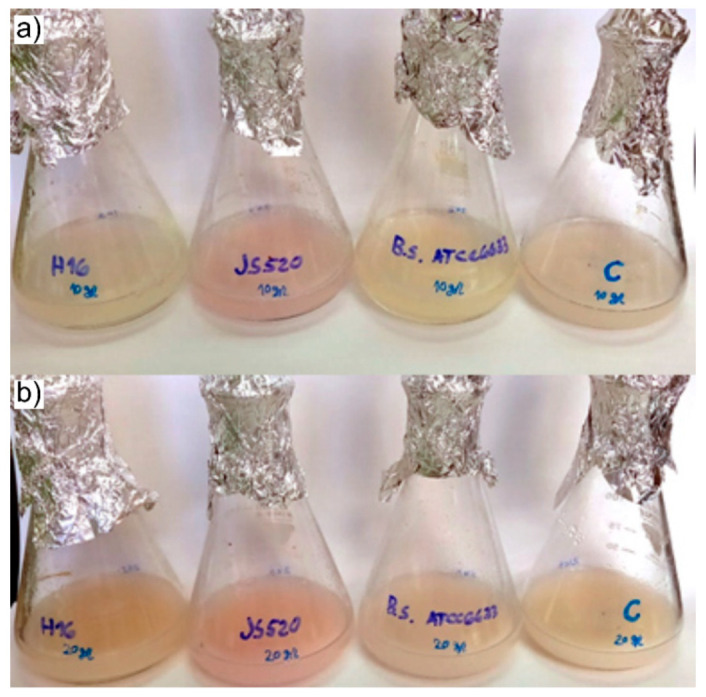
Cultures of *Ralstonia eutropha* H16, *Streptomyces* sp. JS520, *Bacillus subtilis* ATCC6633, and uninoculated control (left to right) in MSM medium with dissolved PVA/TPS material as the sole source of carbon: (**a**) 10 g/L and (**b**) 20 g/L.

All of the cultures were able to use PVA/TPS films as the sole carbon and energy source in liquid culture (Figure 4). Biomass yields were low to moderate with conversion rates between 8% and 14.5% when 10 g/L of PVA/TPS substrate was used (Table 3). Conversion rates were 1.3–1.8-fold higher when double the amount of substrate was used, with *Streptomyces* sp. JS520 reaching a conversion rate of 22% (Table 3). This strain was able to produce purple biopigment, undecylprodigiosin, under both conditions (Figure 4) to up to 5.3 mg/L of culture (Figure S2). 

The *Streptomyces* JS520 strain was previously reported to produce undecylprodigiosin under optimized conditions of 67 mg/L of culture [38]. Although 12.6-fold lower levels of this biopigment were reached in this study, this is the first report of the upcycling of PVA/TPS polymeric materials to biopigments, and further optimizations of the bioprocess could reach higher titers. Undecylprodigiosin and other pigments of the prodigiosin-family have been shown to exhibit multiple bioactive properties, including anticancer [33,59], and are of high commercial value (approx. EUR 360/mg from Sigma-Aldrich supplier) [60]. Previously, fructose and sucrose were found to stimulate the production of undecylprodigiosin in *Streptomyces* spp. [61], while solid substrates such as wheat bran and rice husk stimulated its production in solid-state fermentation [62]. Recently, bioprocessed chitinous marine waste was used for the production of pigment from the prodigiosin family [63].

On the other hand, *R. eutropha* H16, a known PHB producer, showed the least ability to grow on a PVA/TPS carbon source (Table 3) but was also able to accumulate PHB up to 8% of cell dry weight (data not shown). This is considered a moderate to low yield for a biopolymer, but it is the first time that the production of PHB from a PVA/TPS blend has been reported, and, as in the case with undecylprodigiosin, further optimization of the bioprocess would afford higher titers of PHB. The low biomass yields could be due to the fact that wild-type *R. eutropha* H16 lacks gene coding for amylases or glucoamylases, preventing the direct utilization of starch by this strain [64]. Therefore, the obtained biomass and PHB could be due to the utilization of PVA monomers. Nevertheless, saccharified waste potato starch can be used as a carbon source by *Ralstonia eutropha* NCIMB 11,599 to obtain high yields of PHB [31]. Production of PHB from starch by the recombinant *Corynebacterium glutamicum* strain was also demonstrated to yield 6.4% [65], and that from engineered *E. coli* SKB99 yielded 57.4% of cell dry weight [66]. 

The *Bacillus subtilis* strain was included in this study, due to the established production of extracellular α-amylase that efficiently hydrolyzes the starch [67]. This ability could actually be coupled to afford two-stage bioproducts such as biopolymers or biofuels accumulation [68]. Indeed, *B. subtilis* ATCC6633 achieved good growth on PVA/TPS and the highest growth on hydrolyzed PVA (Table 3, Table S1). In fact, all three strains could utilize hydrolyzed PVA as the sole carbon and energy source (Table S1, Figure S3). *B. subtilis* ATCC6633 and *Streptomyces* sp. JS520 were very efficient (substrate-to-biomass conversion of 53% and 76%, respectively) and JS520 could also produce undecylprodigiosin pigment from this carbon source (Figure S3). 

#### 3.2.2. PVA/TPS Degradation in Model Compost

**Figure 5:**
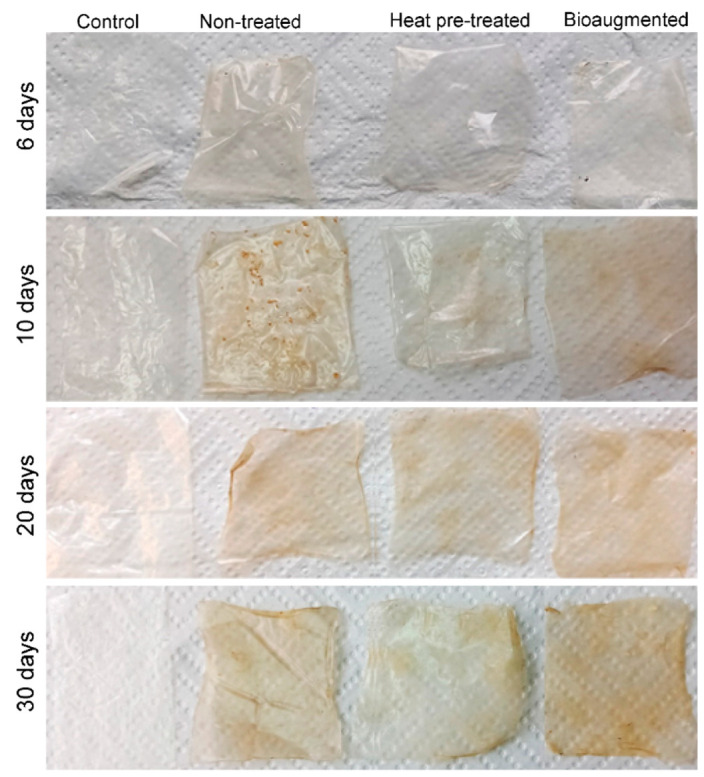
PVA/TPA film samples after compost burial at different time points.

The biodegradability of PVA/TPS films was tested in a model compost system at 37 °C. After 6 days, no change was observed except shrinking and the loss of elasticity. After 10 days, a change in color was observed along with visible cracks in the nontreated and bioaugmented compost samples, as well as orange circles in the heat-pretreated compost samples. After 20 days, discoloration was even more apparent in all samples (Figure 5). A weight loss of more than 30% was detected in all samples within 10 days, while samples in the heat-pretreated compost lost a further 13% wt over the subsequent 20 days (Table 4). Initially, the most efficient degradation occurred in the bioaugmented compost, suggesting that the addition of bacterial strains with a proven ability to hydrolyze and utilize PVA/TPS can be beneficial for the degradation process. However, upon prolonged incubation, the degradation in bioaugmented and heat-pretreated compost was comparable (Table 4). This may be due to the fact that the initial heat pretreatment of the compost favored sporulating microorganisms such as *Actinomyces* and fungi that required some time to develop. 

Previously, starch films degraded completely within 30–84 days, while cellulose, as a reference material, requires 10 days under the same conditions [24,25]. Notably, these investigations carried out composting at an elevated temperature of approximately 60 °C. More recently, the degradation of PVA/starch blend films by two strains isolated from compost has been reported. Strains *Bacillus* sp. DG22 and *Paenibacillus* sp. DG14 were found to degrade films to a comparable extent observed in the current study (45–75% depending on the type of blend used) [21]. A study investigating the degradation of a PVA/starch/glycerol blend with 20% PVA during composting revealed that the degradation of films was mostly due to starch and glycerol degradation, and that after 45 days of incubation, the PVA content was almost intact [69]. *B. subtilis* and *Aspergillus niger* were selected as amylase-producing microorganisms and used to assess the degradation of chemically modified starch/PVA blend films with different additives. The degradation rates were up to 60% for unmodified starch/PVA films and ranged from 50% to 25% for films with chemically modified starch, glycerol, and citric acid. A different study reported biodegradability (~90%) for 50% starch in PVA samples over 28 days [70]. 

**Figure 6:**
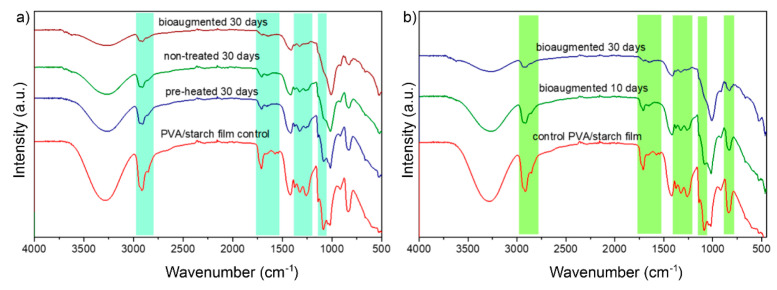
FTIR analysis of (**a**) degraded films after 30 days in different model composting systems, and (**b**) degraded films in bioaugmented composting system over time.

FTIR analysis was employed to detect structural changes in the films over the degradation under different composting conditions (Figure 6). From the FTIR spectrum of the control PVA/TPS film, the presence of a broad peak in the area of 3200–3500 cm^−1^ can be attributed to the vibrational stretching of hydroxyl (–OH) groups, while the peak at wavenumbers between 2780 cm^−1^ and 2980 cm^−1^ was associated with C-H stretching. A sharp peak at 1711 cm^−1^ derived from the carbonyl -C=O functional group, and the low-intensity peak at 1650 cm^−1^ was due to the bound water [71]. The –CH_2_ group characteristic vibrations appeared at wavenumbers of 1420 cm^−1^ and 840 cm^−1^, while the absorption peak at 1300 cm^−1^ derived from the –C–O–C group deformation vibrations. Finally, the peak appearing in the area from 1090 to 1021 cm^−1^ was due to the stretching of aliphatic alcohols [72]. All the peaks visible in the FTIR spectrum of control PVA/TPS films came from both the PVA and starch, as the characteristic peaks of PVA and starch are similar [73,74]; therefore, they overlapped and could not be precisely distinguished. Furthermore, the broad absorption band in the area of 3200–3500 cm^−1^ was due to the inter- and intra-molecular hydrogen bonding of –OH groups in PVA and starch.

After degradation under different composting conditions, the structure of the degraded films was altered according to the FTIR spectra as the intensity of characteristic peaks decreased or completely dissipated, confirming the progressive degradation. The carbonyl peak intensity (at wavenumber of 1711 cm^−1^) after 30 days of degradation significantly decreased in the case of all tested samples (Figure 6a), while in the case of bioaugmented testing conditions, this characteristic peak was hardly visible. A significant decrease in the peak intensity was also detected for the –CH_2_ characteristic peak in the area from 2780 to 2980 cm^−1^. The intensity of the broad absorption band coming from –OH groups decreased, but this peak also broadened, indicating that over the degradation course, the existing inter- and intra-molecular interactions between the –OH group of PVA and starch were disturbed. Further, the intensity of the –C–O–C characteristic band at the wavenumber of 1300 cm^−1^ decreased after degradation under nontreated and autoclaved conditions, while in the case of bioaugmented testing conditions, this peak disappeared. Decreasing peak intensity was also confirmed for the peaks in the area from 1090 to 1021 cm^−1^ (stretching vibrations in aliphatic alcohols) and at the wavenumber of 840 cm^−1^ (–CH_2_ group vibrations). Comparing the degraded films under the different conditions, it could be assumed that films tested under bioaugmented and nontreated conditions indicated greater changes in structure in comparison to the PVA/TPS film tested under preheated conditions. Following the structural changes in the films observed over time from the representative FTIR spectrum (Figure 6b), it could be concluded that changes in the structure were more prominent over time. This was confirmed by the increase in weight loss, decrease in peak intensity, as well as dissipation of the characteristic peak after 30 days of degradation. All the characteristic peaks that were changed over time, decreased in peak intensity, or dissipated, under different testing conditions, changed in the same manner for the films degraded under bioaugmented conditions, whereas the most prominent changes in the vibrations were observed at 2780-2980 cm^−1^ (–CH_2_ vibrations), 1090 cm^−1^, and 840 cm^−1^ (–CH_2_ band vibrations).

**Figure 7:**
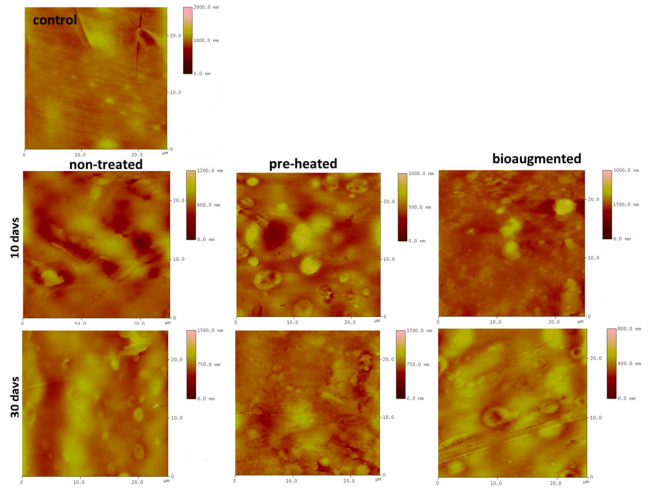
2D AFM images, (25 × 25) μm, of PVA/TPS films before degradation (control) and after 3, 10, and 30 days of degradation under different model composting conditions.

The AFM micrographs of the control film and films composted under different conditions over time are presented in Figure 7. The polymer chains of PVA and starch in control samples formed basal planes due to the hydrogen bonds between those two hydrophilic polymers, and therefore, a planar surface with no irregularities was observed (light yellow color).

The surface morphology was significantly altered after degradation in all three tested media, which was indicated by darker areas, representing higher elevations from the base of degraded films. Visual changes observed from 2D AFM images were supported by changes in surface roughness (RMS) measurements (Table 5). In the starting 10 days of degradation, RMS values increased in all three tested media with respect to the control sample (74.6 m), confirming surface erosion caused by enzymatic attack. After 30 days of composting, the RMS values were lower than those calculated for the 10 days of composting, but higher than those for the control sample for composting under preheated and nontreated conditions. This phenomenon could be explained by the starting erosion of the films that resulted in the increase in RMS values followed by the greater weight loss after 30 days of degradation when the films’ surface was smoother as the thin layer of the films was eroded and the degradation products mostly leaked out of the remaining polymer matrix.

**Figure 8:**
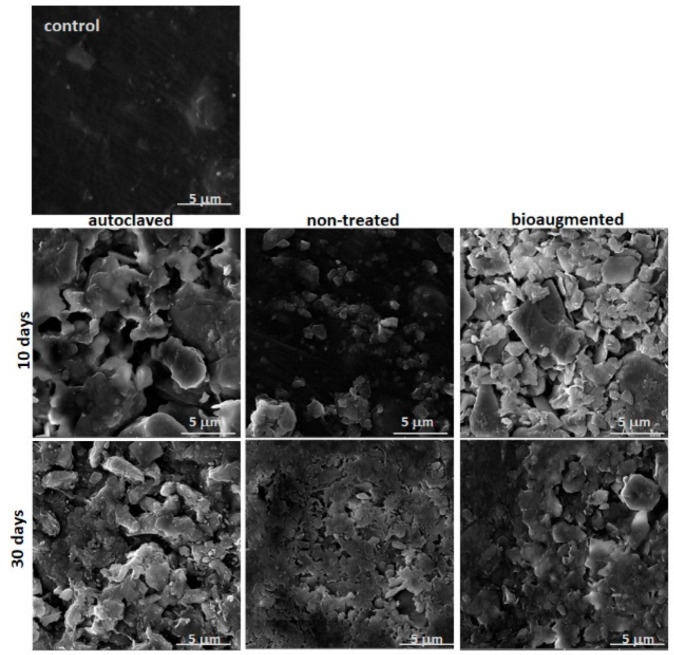
SEM images of PVA/TPS films after 10 and 30 days of composting under different conditions (magnification of 10,000×).

In order to gain an insight into the changes in morphology caused by the activity of enzymes presented in different composting systems, SEM analysis was also carried out and the recorded images of the PVA/TPS films before and after degradation are shown in Figure 8. 

Pure PVA films appeared to have a planar, smooth surface and seemed to only possess visible agglomerates and granules after blending with starch [55]. Images of the control PVA/TPS film in Figure 8 indicated quite a smooth and planar surface morphology, with no visible signs of starch particles. After a degradation period of both 10 and 30 days, remarkable changes in surface morphology were detected. While those changes were less prominent in the case of films composted under nontreated conditions after 10 days, PVA/TPS films tested under preheated and bioaugmented conditions were highly disintegrated, and a lot of cracks and holes appeared on the surface. Observation of SEM images for the degraded samples clearly showed disintegration across entire films on the surface, indicated by a plethora of small crumbled/fractured parts, although the material strips remained somewhat consolidated overall but with significant weight loss. These results all point to the surface degradation mechanism so common for the enzymatic degradation that is, due to the activity of enzymes, excreted by the microbes present in the compost.

**Figure 9:**
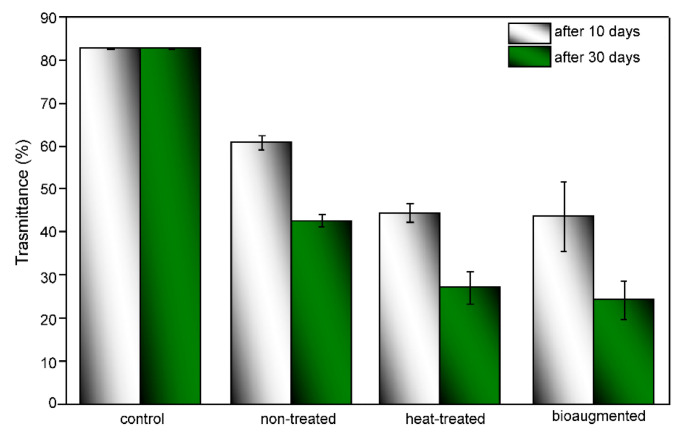
Changes in the % transmittance of the composted PVA/TPS films over time in the visible region from 200 to 400 nm.

One of the desired properties of packaging materials is light barrier properties, especially for UV radiation. Light barrier properties of the PVA/TPS films before and after composting under different conditions were observed in terms of the % transmittance (% *T*) for wavelengths in the range from 200 nm to 800 nm. As the % transmittance of investigated films in the UV region (200–400 nm) changed during degradation while the % transmittance remained constant in the visible region, changes in the % transmittance in the 200–400 nm UV region after 10 and 30 days of composting were calculated and are presented in Figure 9. The obtained results were in agreement with the captured photos of investigated films shown in Figure 5. In order to minimize the effect of compost material that might remain on the films due to degradation, which would further affect % transmittance measurements, the degraded films were washed with cold water. After 10 days of composting, the % *T* of preheated and bioaugmented samples decreased from 80% to approximately 45%. Over the same period, the decrease in % *T* for the nontreated sample was smaller, from 80% to 65%. The % *T* of the preheated and bioaugmented samples after 30 days was significantly reduced in comparison to the control sample, and decreases in % *T* of 44% and 27%, respectively, were detected. Changes in the % *T* were in line with the visible appearance of composted films (Figure 5), as after the degradation, PVA/TPS films became less transparent (Figure 9).

## 4. Conclusions

In this study, low-energy, low-carbon post-use upcycling was demonstrated and presented as a model approach for PVA/TPS material circularity. The PVA/TPS film mechanical properties were comparable with those of LDPE and, hence, represent one of the improved mechanical performance and moisture sensitivity blends that have been developed recently [75]. Importantly, this work pioneers the conversion of the PVA/TPS film substrate to a high-market-value biopigment (undecylprodigiosin) at 5.3 mg/mL and/or PHB biopolyester at 7.8% of cell dry weight. The high degree of hydrophilicity and its solubility in water render this material an accessible carbon source for bioprocessing. Film amenability to biodegradation within compost systems was also demonstrated, with more than 50% weight loss occurring after 10 days at 37 °C. The upcycling approach described in this study has the potential to be instrumental in mitigating the damaging socioeconomic and environmental impacts of plastics production, increasing unabated, expected to double over the next 20 years.

## Figures and Tables

**Table 1:** Summary of tensile test results, including tensile strength (*σ*_u_), strain at break (*ε*_u_), and Young’s modulus (*E*).

	Cast	Extruded: Longitudinal	Extruded: Transverse
*σ*_u_ (MPa)	37.2 ± 2.2	38.6 ± 4.6	22.2 ± 4.3
*ε*_u_ (%)	197 ± 70.0	136 ± 17.0	325 ± 73.0
*E* (GPa)	249.9 ± 94.7	60.3 ± 20.4	52.9 ± 14.1

**Table 2:** Range of PVA/TPS mechanical properties compared to those for typical polyethylene film materials (low-density polyethylene (LDPE), high-density polyethylene HDPE, linear low-density polyethylene (LLDPE)) [51], PVA [52], and TPS [53].

Property	PVA/TPS	LDPE	HDPE	LLDPE	PVA	TPS
*σ*_u_ (MPa)	22–39	36–57	38–44	36–60	22–30	4–8
*ε*_u_ (%)	136–325	300–500	600–860	450–850	99–112	35–100
*E* (MPa)	53–250	190–520	827–1069	204–275	64–176	116–294

**Table 3:** Biomass yield (mg dry mass/mL of culture) and conversion rate from bacterial cultures grown in MSM medium containing 10 and 20 g/L of PVA/TPS film.

	MSM + PVA/TPS Film 10 g/L		MSM + PVA/TPS Film 20 g/L	
Strain	Biomass, mg/mL	Conversion, %	Biomass, mg/mL	Conversion, %
*Ralstonia eutropha* H16	0.80 ± 0.08	8	2.75 ± 0.05	13.7
*Streptomyces* sp. JS520	1.20 ± 0.09	12	4.40 ± 0.08	22.0
*Bacillus subtilis* ATCC6633	1.45 ± 0.04	14.5	3.95 ± 0.04	19.7

**Table 4:** Weight of washed and dried pieces of plastic bag after compost degradation. The weight of pieces prior to burial was approximately 38 mg.

Compost	Day 3	Day 10	Day 30	Weight Loss, %
Nontreated	31 ± 2 mg	22 ± 1 mg	22 ± 1 mg	42
Heat-pretreated	29 ± 1 mg	25 ± 2 mg	20 ± 1 mg	47
Bioaugmented	23 ± 1 mg	22 ± 1 mg	19 ± 1 mg	50

**Table 5:** AFM analysis results: RMS values of degraded films.

Sample *	RMS, nm 3 Days	RMS, nm 10 Days	RMS, nm 30 Days
PVA/starch film nontreated	104.7	100.3	98.8
PVA/starch film heat-pretreated	195.5	91.5	77.4
PVA/starch film bioaugmented	130.8	150.7	66.5

* RMS value of control sample was 74.6 nm.

## Data Availability

The data are available on contact with the corresponding author.

## Supplementary Materials

The following are available online at https://www.mdpi.com/article/10.3390/polym13213692/s1, Figure S1: Experimental setup of biodegradation under composting conditions: (a) model compost in Petri dishes, (b) PVA/starch film in model compost; Figure S2: UV-Vis spectrum of the extracted undecylprodigiosin from *Streptomyces* sp. JS520 grown in MSM medium containing 10 g/L of PVA/TPS substrate and the appearance of the EtOAc culture extract; Figure S3: Pellets of centrifuged cultures grown in MSM medium with (A) 5 g/L of PVA and (B) 10 g/L of PVA. From left to right: *Ralstonia eutropha* H16, *Streptomyces* sp. JS520, and *B. subtilis* ATCC 6633; Table S1: Biomass yield (mg of dry mass/mL of culture) and conversion rate from bacterial cultures grown in MSM medium with PVA as sole carbon source.
