# Supplementary material for: Upcycling Biodegradable PVA/Starch Film to a Bacterial Biopigment and Biopolymer
Source: Polymers (Basel). 2021 Oct 27;13(21):3692. doi: 10.3390/polym13213692 (PMC8588134; doi:10.3390/polym13213692)
Supplement: Supplementary file 1 [file polymers-13-03692-s001.zip › polymers-1372524-supplementary.pdf]

Supporting Information

Upcycling Biodegradable PVA/Starch Film to a Bacterial Biopigment and Biopolymer

Brana Pantelic[1#], Marijana Ponjavic[2#], Vukasin Jankovic[1], Ivana Aleksic[1], Sanja Stevanovic[2], James Murray[3], Margaret Brennan Fournet[3] and Jasmina Nikodinovic-Runic[1*]

*Corresponding author:

Tel. +381 11 3976034; E-mail: jasmina.nikodinovic@imgge.bg.ac.rs

Fax: +381 11 3975808; Vojvode Stepe 444a 11000 Belgrade, Serbia

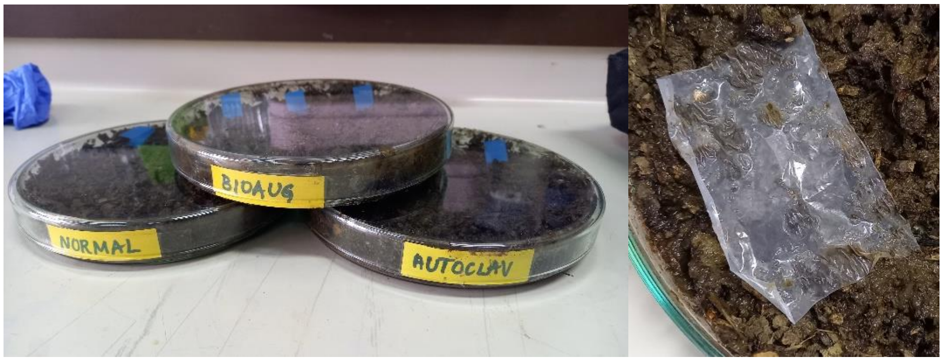

Figure S1. Experimental setup of biodegradation under composting conditions: model compost in Petri dishes (left) and PVA/starch film in model compost (right).
<u>Model compost characteristics: </u>pH 7.08; $CaCO_3$ – 1.23%, Nitrogen- 0.65%, P_2O_5 – 219 mg/100 g; K_2O – 244 mg/100 g

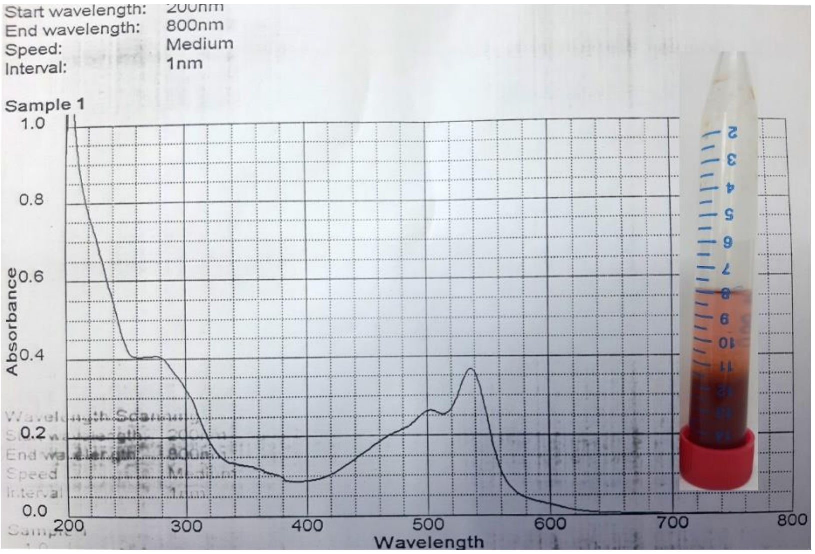

Figure S2. UV-Vis spectrum of the extracted undecylprodigiosin from *Streptomyces* sp. JS520 grown in MSM medium containing 10 g/L PVA/TPS substrate and the appearance of the EtOAc culture extract.

Table S1. Biomass yield (mg of dry mass/ mL of culture) and conversion rate from bacterial cultures grown in MSM medium with PVA as sole carbon source.

	MSM + PVA 5 g/L		MSM + PVA 10 g/L	
Strain	Biomass, mg/ml	Conversion,%	Biomass, mg/ml	Conversion,%
Ralstonia eutropha H16	1.25 ± 0.06	25	2.75 ± 0.05	27
Streptomyces sp. JS520	2.65 ± 0.06	53	7.20 ± 0.04	72
Bacillus subtilis ATCC6633	3.80 ± 0.04	76	7.00 ± 0.08	70

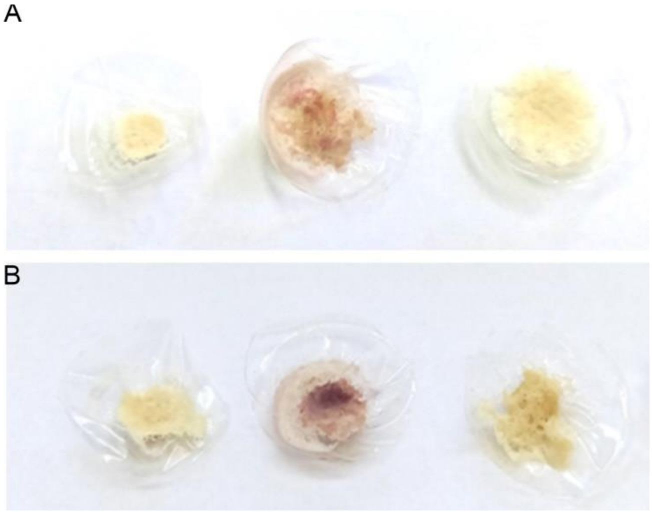

Figure S3. Pellets of centrifuged cultures grown in MSM medium with: A) 5 g/L of PVA and B) 10 g/L of PVA. From left to right: *Ralstonia eutropha* H16, *Streptomyces* sp. JS520 and *B. subtilis* ATCC 6633.
